# It's just how we do it: social processes in rapid weight loss for combat sports

**DOI:** 10.1080/21642850.2024.2433517

**Published:** 2024-11-26

**Authors:** Susan J. Wilbraham, David Elliott, Paul K. Miller

**Affiliations:** Institute of Health, University of Cumbria, Cumbria, UK

**Keywords:** Rapid weight loss, social psychology, health risks, combat sports, making weight

## Abstract

Making weight is an essential aspect of preparation for competition in combat sports. In addition to gradual weight loss in order to fight lean, fighters commonly engage in Rapid Weight Loss (RWL) practices seeking short-term hypohydration in order to be lighter for weigh-in. The aims of this study were to explore the RWL-related experiences of *N* = 7 participants in non-professional UK Thai boxing (Muay Thai), in order to elucidate the socio-cultural and social-psychological processes relating to these behaviours. Four themes were identified: One-upmanship; Setting and conforming to expectations; Self-directed preparation; and Subversion of RWL controls. These themes describe how RWL is learned, rewarded, magnified, often unmitigated, and inherently difficult to restrict. Contrary to suggestions in past research, participant accounts highlight how fighters may aggressively cut weight at lower levels of the sport where health-related interventions are harder to implement.

## Introduction

Prospectively hazardous weight-management practices have been of considerable interest to health psychologists for some decades (Carter et al., 2023; Cooper et al., 2007; Meneguzzo et al., 2021). Within the corpus of contemporary research addressing this broad issue, clinical eating disorders are the central focus for a clear majority of such studies, given (a) their widespread occurrence, and (b) their complex social and psychological antecedents and impacts (Berge et al., 2012; Davey et al., 2023; Tuval-Mashiach et al., 2014). Similarly, issues surrounding ‘fad’ dieting (Mann & Nye, 2009; Tahreem et al., 2022) and compulsive exercising (Cuesta-Zamora et al., 2021; Kolnes & Rodriguez-Morales, 2016) are well documented. However, one weight-management technique that has received less attention in the broad field of health psychology is Rapid Weight Loss (RWL) in otherwise healthy individuals. RWL refers to the prospectively hazardous practice of aggressively decreasing body mass to meet a specific, and usually short-term, weight criterion (Alwan et al., 2022; Lakicevic et al., 2022; Neighbors & Sobal, 2008). While not uniquely so, this custom is especially prevalent in combat sports such as boxing, Olympic-style wrestling, judo and mixed martial arts (henceforth MMA), where weight classifications are a fundamental component of the combat environment (Artioli et al., 2010; Artioli et al., 2016; Lakicevic et al., 2020; Matthews et al., 2019; Roklicer et al., 2020; Roklicer et al., 2022).

Weight classifications in combat sports are manifestly designed to render bouts fairer and safer, reducing the potential for serious injuries borne of substantial differences in strength, body mass and power (Matthews et al., 2019). As such, competitors are weighed before a fight such that they can qualify for a particular weight class, or pre-agreed fight weight. However, it is known that many combat athletes, will aggressively ‘cut weight’ in the three to seven days before weigh-in, in order to qualify for a match with a lighter, and thus theoretically ‘easier’, opponent (Crighton et al., 2016; Lakicevic et al., 2020). They will then, between weigh-in and bout, endeavour to rapidly regain as much weight as necessary to gain a perceived advantage. The amount of weight lost in such cases has been demonstrated to typically range between 2.5% and 8.5% of habitual body mass (Brito et al., 2012; Connor & Egan, 2019), although losses of 10% or more have been reported for some time (Artioli et al., 2016; Steen & Brownell, 1990).

### The scale and impact of the practice

RWL behaviours have been shown to be highly prevalent in combat sports with some studies suggesting that between 60% and 90% of participants engage in such behaviours (Artioli et al., 2010; Brito et al., 2012). In addition, it is known that such practices are far from confined to the elite domain (Crighton et al., 2016; Samadi et al., 2019). Given the increasing popularity of combat sport participation, thus, RWL has the potential to become a significant public health issue. Although reliable contemporary statistics are difficult to assemble, Sport England has recently calculated that by 2022, 827,000 adults were participating in combat sports at least twice per month in England – where the current research was undertaken – alone (Sport England, 2023). If the most conservative of the research approximations are correct, this would suggest that nearly half a million individuals of 16 years and over could potentially be involved in – or at least exposed to – RWL practices. Troublingly, there is strong evidence that under-16s also routinely engage in RWL related to combat sports (Berkovich et al., 2016; Dubnov-Raz et al., 2016; Lakicevic et al., 2020; Lakicevic et al., 2022), though rates of incidence are more difficult to ascertain.

Evidence pertaining to the broader and/or long-term health risks of recurrent RWL in combat sports is somewhat mixed, and a matter of considerable dispute in some academic sectors (Artioli et al., 2016; Artioli et al., 2017; Davis, 2017). There is little doubt, however, that the endemic practices can strain the physiological and psychological well-being of those involved. In the most extreme cases, RWL practices have clearly contributed to fatalities. For example, in December 2015, 21-year-old flyweight MMA fighter Yang Jian Bing died following his attempts to make weight (Reinsmith, 2017). Further media-noted RWL-related deaths have included those of Jessica Lindsay and Jordan Donald (Campbell, 2017). While these might be viewed as isolated incidents, and RWL practices are indeed seldom fatal, it is significant that these deaths have paralleled strong calls from within the sport and nutritional sciences for such practices to be banned (Artioli et al., 2016; Crighton et al., 2016; Lakicevic et al., 2022).

### RWL in combat sports: what are the practices and risks?

The core RWL techniques involved in combat sports have been typically reported to involve the restriction of food (particularly carbohydrates) and active hypohydration of the body. Calorie restriction involves fasting and/or the reduction of specific food intake (Brito et al., 2012). Instructive research around combat sports indicates that a large majority of participants continue to employ such methods (Artioli et al., 2010; Connor & Egan, 2019; Franchini et al., 2012; Park et al., 2019), despite robust evidence that this practice may engender cognitive impairment and mood disturbance, notwithstanding prospective reductions in physical performance (Aloui et al., 2016; Isacco et al., 2020; Malcolm et al., 2024). It is, however, the issue of hypohydration that is of key concern in this paper, given that food restriction alone cannot typically induce the order of rapid weight loss typically employed within the timeframe pertinent to combat sports.

Short-term hypohydration can be achieved through a number of practices including direct water restriction, the use of diuretics and laxatives, forced vomiting and forced perspiration (steam rooms and heavy exercise in nonpermeable clothing are common means to the latter). Also reported in this domain has been the use of chewing gum to hyper-stimulate the production of saliva (which is then spat out), and ‘water loading’, where competitors (counter-intuitively) drink large quantities of water to promote disproportionate urine production (Artioli et al., 2010; Barley et al., 2018a; Barley et al., 2018b; Crighton et al., 2016; Dube et al., 2022; Matthews & Nicholas, 2017). Hypohydration remains accepted practice in many combat sports, allowing athletes to be lighter at the weigh-in, though the associated techniques can themselves have serious adverse health impacts. Before a fight even begins, for example, the practice of water loading has been compellingly associated with the potentially life-threatening condition of hyponatremia (Knechtle et al., 2019; Verbalis et al., 2007), and an elevated risk of kidney damage (Drid et al., 2023; Trivic et al., 2023). Recent research has reported that 57% of UK MMA fighters utilise this technique (Matthews & Nicholas, 2017), while an Australian study indicates an even greater incidence (Barley et al., 2018a).

A further hypohydration-related risk to a fighter's health, pre-bout, emerges because of hyperthermia, most commonly associated with attempts to achieve dehydration via perspiration. The body natively responds to internal heat from high levels of exercise, and/or environmental heat, by increasing blood flow to the skin's surface and by sweating (Chapelle et al., 2020). Where the necessary evaporation of sweat for systemic cooling is inhibited, the amount of heat gained by the body can exceed the heat lost, further raising the core temperature; in extreme cases, typically at 40.5°C or more, this can become life-threatening (Barley et al., 2018b; Chapelle et al., 2020; Dube et al., 2022). High external humidity (such as in a steam room), sweat being wiped away (common in rigorous exercise), the wearing of non-breathable clothing and, particularly, a combination of these can work to precipitate optimal conditions for a hyperthermic event. Hyperthermia was noted as a cause of death for a college wrestler who died following RWL (CDC, 1998).

Key research has illuminated that while the negative impacts upon immediate brain volume caused by hypohydration practices (if not severe) may be largely or entirely reversed through rapid rehydration, a significant proportion of attempts at this rehydration are not necessarily successful (Barley et al., 2018b; Duning et al., 2005; Trangmar & González-Alonso, 2019). Jetton et al. (2013), for example, document that of the MMA fighters they surveyed, competing at a variety of levels, 39% reported having significant or serious hypohydration-related cognitive and/or somatically functional difficulties immediately before competition. It should also be noted that such hypohydration is definitively associated with reduced blood plasma volume which, in turn, reduces maximal cardiac output. As the heart must work harder to circulate blood, this ultimately reduces baseline endurance (Barley et al., 2018b), and the same physiological mechanism is further understood to account for reductions in aerobic power (Trangmar & González-Alonso, 2019) and active musculoskeletal weakening/damage (Roklicer et al., 2020); in short, the hypohydrated fighter is likely to be underpowered and more easily fatigued, thereby increasing their chances of producing inferior performance and sustaining injury. This is of particular concern, given that particularly higher degrees of hypohydration can also cause an accumulation of cerebral spinal fluid around key ventricles of the brain (Kempton et al., 2009; Zhang et al., 2022), rendering the brain itself more susceptible to impact forces and enlarged risk of cerebral contusion (Besenski, 2002).

While some competitors may fight whilst still moderately or even severely hypohydrated, others may not fight at all as a consequence of it. Heat illness from RWL can cause competitors to withdraw due to symptoms such as dizziness, low blood pressure, syncope (blackouts), nausea, vomiting, cramps or seizures (Brignole et al., 2018; Weber et al., 2013). In addition, RWL via water eradication can impact the psychological state, with abundant evidence indicating that even relatively mild levels of hypohydration can lead to reductions in vigilance, visual working memory and response latency, and increases in tension, anxiety and pain experience (Dube et al., 2022; Moyen et al., 2015; Weber et al., 2013). For an individual entering an intense fight context, to be compromised in terms of concentration, fatigue and response latency somewhat inevitably increases the chance of sustaining serious harm (Artioli et al., 2016; Lakicevic et al., 2022; Rossi et al., 2022).

### The social context(s) of RWL

It is clear that the bulk of research in sports science addressing combat sport-related RWL, prior to – and even after – the emergence of contemporary well-being concerns, primarily addresses the equation between performance benefits and physical risks (Artioli et al., 2017; Davis, 2017). Comparatively little investigation has explored the social-psychological processes that maintain the ‘arms race’ of RWL. This reflects the dynamic in which participants undertake a set of behaviours that are not only designed to gain an advantage but to avoid losing one; i.e. both competitors can reasonably be assumed to be doing the exact same thing. It is in this respect that the intervention of Pettersson et al. (2013) provides an important counterpoint. Using a Grounded Theory model, the authors forensically highlight the specific and personalised experiences of ten fighters in elite combat sports (in Sweden), all of whom engage with RWL. Key findings indicate that the participants view RWL as, firstly, a means of developing fighting self-efficacy through a broader sense of self-discipline. Secondly, they use it as a diversionary tactic around negative thoughts and worries pre-bout. Thirdly, RWL is considered an essential part of their identity within their sport; i.e. it engenders a sense of belonging. Ultimately, fighters viewed RWL as being so closely linked to the traditions and culture of the sport that they had themselves become an integral (and largely unquestioned) part of that culture.

The work of Pettersson et al. (2013), while critical in developing a more nuanced understanding of athletes’ motivations to take part in RWL practices, nevertheless ultimately arrives at something of an intentionalist, uses-and-gratifications model of individual behaviour (Ruihley et al., 2022). While the endemic threats posed by RWL to the health of fighters are well noted in their research, Petterson and colleagues take more limited account of how the culture in question is understood, built and ultimately reproduced by those involved in it. This order of socio-cultural concern has subsequently been explicitly addressed within a qualitative account of team-doctoring in Muay Thai (Al-Hashmi & Matthews, 2022). Findings herein acknowledge the cultural embeddedness of lay sports medicine, the complexity of the symbolic interactions that shape communication, and the acceptance and internalisation of practice, including (though not explicitly focusing upon) that of weight cutting.

The work detailed below builds on these previous accounts by focusing on the RWL subculture and its relationship with the psychology of health. In the business of this, the current study seeks not only to elucidate the experiences and perspectives of fighters themselves, but also those of individuals involved in the upward chain of training, management and promotion. As such, this paper qualitatively explores the experiences and positions regarding RWL of key agents in the UK involved specifically in Muay Thai. Analysing extended semi-structured interviews using the model of thematic analysis outlined by Braun and Clarke (2006, 2019), accounts provided by participants are explored to generate a multi-perspective, locally relevant interpretation of the culture of RWL. This, it is contended, takes steps towards providing a broader understanding of the social-psychological pressures on individuals involved in combat sports to sustain RWL practices, and the difficulties that might emerge in effecting behavioural change at the collective level.

## Method

### Participants

Ethical approval was obtained for this research from the University of Cumbria ethics panel (ref 15/ 56) and informed consent was gained from all participants. Participants were purposively selected via social media advertising, given (a) their (self-identified) experience in the field, and (b) their varied positions within it. All recruited participants (*n* = 7) were active in Muay Thai in England at the time of recruitment, and in a range of different (and sometimes multiple) capacities, though none had access to club-affiliated medical support, and all had other paid jobs. Participants’ roles are summarised in Table 1.

**Table 1.** Summary of participants’ roles in Muay Thai.

Participant number	Roles
P1	Coach, event promoter, competitor
P2	Coach, judge, competitor
P3	Competitor
P4	Competitor
P5	Competitor
P6	Club owner, coach
P7	Competitor

Some participants had trained in additional combat sports such as Ju Jitsu, Mixed Martial Arts (MMA) and Tae Kwon Do, providing an additional layer of perspective on combat sports in general. Conditions of ethical approval preclude the detailing of any further demographic information, including ages, years of experience or gender, due to the high potential for recognition within a close-knit sporting community.

### Procedure

Extended semi-structured interviews were conducted in person by the first author, to explore participants’ experiences of RWL. A discussion was opened around two core issues, and participants were asked to elaborate on each, providing examples from practical experience wherever possible. These were:

1. Can you describe your experiences of weight cutting in your sport?
2. What is your position on weight cutting, and why?

No additional prompts were required; interviews lasted between 28 and 80 min, with a mean duration of 50 min. Interviews were audio recorded and subsequently transcribed in full. To protect the identity of participants, in line with conditions of ethical approval, transcripts were redacted of all genders, proper names, locations, dates and any other key nominal matters that might reveal specific identities within a prospectively close-knit community. In the extracts presented, ‘ … ’ denotes that text has been removed to these ends, and/or for clarity of presentation.

### Analysis

Analysis proceeded in line with the reflexive precepts outlined by Braun and Clarke (2006, 2019). Provisional coding was conducted by the first author, an experienced chartered psychologist, and then redeveloped with the third, a veteran health researcher. The second author, a seasoned psychologist with extensive experience in research in the sport and exercise field, further revised the process until all three authors found a triangular consensus (Miller et al., 2015) around the analytic product. At this point, the major themes were worked up by all three authors, as documented below.

### Trustworthiness

The trustworthiness of the research was carefully addressed to comply with the standards set out by Yardley (2000), where not otherwise described in this section. *Transparency and coherence* were maintained through the correspondence between clearly available data and its interpretation. *Impact and importance* of the study were tested through the presentation of findings by the first author at an international conference of researchers and practitioners, prior to final writing. In addition, a peer review of the paper was undertaken by a psychologist with direct experience of competing in Muay Thai, and the feedback received has been incorporated into the final analysis.

## Findings

Four major themes were identified in the data, these describe the social processes involved in RWL behaviours from the perspectives of the involved participants:

1. One-upmanship
2. Setting and conforming to expectations
3. Self-directed preparation
4. Subverting RWL control(s)

1. One-upmanship

The first major theme describes participants’ routine contention that RWL provides a weight advantage which, in turn, translates into a competitive advantage. It should be noted for context that in some competitions, fighters will apply for a contest in a specific weight category. In others, however, the fight weight is set through negotiation between the fighters’ club(s) and a contest organiser.

You try and be the very heaviest you can be in the next weight category down, so that gives you an advantage cos you’re going to be heavier and probably stronger. [P1]

Like for me it's an advantage because I’ll always fight someone a little bit smaller … I see it as an advantage and would be gutted if the advantage were taken away from me. [P7]

This position was not taken naively by most participants. Indeed, some very explicitly noted that they understood both fighters in a contest would be aiming to gain the exact same advantage and, moreover, that this could engender a shifting of focus from the bout itself to simply being ‘better’ at RWL than the opponent.

It's not a dieting competition, it's a fighting competition. It's who is more skilled at that sport … I don't want to get good at [weight-cutting] … I don't feel like I need to bother with this try and be a pro dieter type thing. [P3]

In a similar vein, P6 drew attention to the prospective futility involved when fighters of equivalent weights engage in equivalent levels of weight-cutting:

If you’re both taking your fight at 64 kilo, and you’re both 70 kilo on Monday, and you both do a six kilo weight cut in that week, you may as well both have not bothered.

In practice, however, it was commonly identified that fighters who do not engage in RWL practices typically do find themselves at an active in-bout disadvantage, deepening the pressure to supersede the presumed weight-cutting activities of their opponent:

We always end up fighting somebody heavier but that's just one of the things we do I suppose … I think it does put us at a disadvantage. [P5]

Some clubs don't weight cut. They always find it as a disadvantage when they fight … If you don't weight-cut and your opponent has, you’re going to be smaller, he's going to be bigger than you. You don't want to fight anyone who's bigger and stronger. [P7]

2. Setting and conforming to expectations

The socio-cultural pressures to which fighters are routinely exposed emerged as a major theme in participants’ accounts. It was particularly widely reported that (a) there was an order of reverence among those involved in Thai boxing regarding (a version) of its lore and history, which could exert an influential cultural pressure, and that (b) more acculturated others (e.g. peers, coaches, clubs, and/or contest organisers) could exert more direct pressure via instruction, encouragement and advice.

(2a) Culture of Thai boxing

Participants documented this culture in terms of both its Thai origins and its ingrained normativity within the contemporary Thai boxing industry:

In Thailand it's just more old school … it's just a very old school way of doing it, it's just like how light can you fight? [P7]

I think people in the Thai boxing industry, they’re just very stubborn … .to persuade them to say weight cutting maybe isn't brilliant for you after all the years of them doing it. It's like telling them like the roundhouse kick, the most famous kick in Muay Thai is wrong. [P5]

A sense of competitiveness around RWL itself was commonly viewed (for better or worse) as a cornerstone of the modern Muay Thai ethos, with the struggle to make weight a celebrated part of the process, and its achievement a mark of authentic commitment to the sport.

I think there probably is a bit of swagger about ‘Oh I’ve got to cut eight kilos’, and the feeling of triumph when you have done ‘cos it's so difficult. [P6]

It's horrendous, it's not a nice thing to do. But I think that once they come out of it then they’re glad it's over and they feel like an accomplishment … I get a massive buzz out of making that weight … you go: ‘By this time, I’m gonna weight 82 kilos, I’ll set my target, watch me right’, and everyone's going, ‘Bollocks you’re 90-odd kilos’ … I got down to 79 kilos, so I was like … ’Right watch this now, I’m under 80’. [P1]

The centrality of weight cutting in the sport was widely cast as being ‘It’s just how we do it’ [P7], a near-universal cultural feature.

The general consensus because it's been so common for so long is that the weight cut is part of your training, it's part of your fight camp, so if you can tough out a super mega-awful water cut then you deserve to get the advantage. [P6]

The embeddedness of RWL in Muay Thai was, perhaps, best evidenced by participants’ own analyses of how the risk-reward equation was managed by its proponents:

That MMA fighter, I think, unfortunately died as part of his weight cutting … My thought was like: ‘See why would you do it? Someone has just died because they tried to cut weight. Why would you?’ and then that sparked a lot of debate, and people were still like, ‘Look he obviously didn't do it safely’. So, then people started talking about water cutting safely and stuff like that and they’re still defending it. [P3]

While the ubiquity of this wider cultural pressure was addressed by all participants, some viewed the local culture and coaches at individual clubs as ultimately being a stronger influence on fighter behaviour:

If all the coaches said, ‘You’re not water-cutting’, then none of the students would water cut, pretty much. [P6]

At our gym we gradually lose the weight, so we just burn the fat off, we just become completely muscle. We don't do any water-cutting. [P5]

Whatever the participant’s position on RWL itself, however, it was unanimously accepted that there was no meaningful drive to prevent the practice, and that a sense of tradition was key a barrier to change:

The biggest barrier by any stretch is literally just habit and tradition, as in, like, not traditional Muay Thai. [P6]

(2a) Conforming to expectations

At the procedural level, it was broadly accepted by participants that expectations to engage in RWL (and the limits thereof) begin for a fighter at the point where a specific bout is agreed:

I think people need to be realistic. If you walk at 70 you should be fighting no less than 64 kilos, ‘cos it's just ridiculous to go any further than that. [P2]

Some participants further noted that associated pressures are not only placed on the fighter, but also on the fighter’s club owner and coaching staff:

Your credentials really are your fight wins … it's held my gym back and my fighters but I would rather have healthy strong fighters than loads of title belts across the ceiling … My fighter used to get fellas from other gyms which utterly pissed me off, talking to him saying, ‘You know you should be fighting lighter than this’. I’ve been utterly pressured to get my fighters to water cut, and that I’m doing them a disservice, and I’ve had to just ignore that and live with it and just think, ‘I know I’m doing the right thing’. [P6]

Once a fight weight has been agreed, a range of techniques were described by fighters for making weight at virtually any cost, with an attendant anxiety around failure a further driving factor:

You get in the sauna and you just put more clothes on, you know, you just wait, get out, dry, get on, have a look at the numbers, get back in. You don't want to get back in but you get back in … I don't know when I would stop and it's a scary thought but … I need to be at that weight, I need to get there. I don't know what would stop me. [P7]

I didn't trust my scales at home … I went in the sauna cos I felt like that's what I had to do just to make sure. So, I went in for an hour and I lost a kilo and a half in an hour. It was hell … I was like, ‘I don't know how much; I’ll just go in for an hour’. Was the same with the water, I was like, ‘Well I shouldn't drink any water, so I won't’, so I was there just keeping my lips wet and my tongue wet, thinking, ‘Well I can't drink water; I don't know how much weight I’ll put on if I have any water’. So, all of that put together ended up with me being two kilos under the actual weight that I was supposed to weigh-in at. [P3]

Indeed, if fighters do not make weight, they may be explicitly required by a coach (or other significant agent) to engage in further RWL behaviours before returning to the scales.

A girl weighed point-seven kilos over … she had to stand there in a bin bag skipping the point-seven kilos off. [P2]

Making weight (and anxieties around it) were also linked to the matter of ‘professionalism’; failing to make weight would be viewed in the community as unprofessional, a threat to identity. This, in turn, was taken to give those who do make weight a psychological advantage when their opponent does not.

If someone doesn't make weight you try and make them feel bad about it, ‘cos it's like psychological. My opponent didn't make weight, but I accepted the fight anyway’, and I got loads of comments saying, ‘You’ve got a psychological edge now cos you made weight, and he didn't. You look more professional because you made it and he's not’. [P3]

The expectation to make weight was such that participants documented cases where they had supported peers to continue with a weight cut, even where they were unwell.

I literally had to carry one fighter to the weigh-in; it was disgraceful … she hadn't lost the weight gradually, so this is what she felt she had to do, and to watch somebody go through that, and be barely able to walk from the front door to their car, and then have to carry them out to stand on the scales … it doesn't register. [P2]

I wouldn't know what I would do if my mate was in the sauna, and he started feeling dizzy. I wouldn't know what to do. Do you give him water? Do I make him put weight back on by giving him water? Because you’re not allowed to put anything passed your lips, an ice cube, that's the only thing you do, put an ice cube in your mouth. So, what do you do? [P7]

3. Self-directed preparation

The fighters described in this study were commonly unsupported during their preparation for a fight, with the responsibility being entirely (or almost entirely) upon them to manage their attempts to successfully make weight.

3(a) Supervision and monitoring

It was reported that fighters will typically be advised to lose any excess weight during the weeks preceding the competition, so that RWL is kept to a minimum. While coaches may well advocate such gradual weight loss for competition, however, they have little control over (or, indeed, responsibility for) fighters’ wider behaviours.

If you ask for my advice and I give you my advice and follow my advice you’d be on weight. If you leave it too late and then you start talking about cutting weight, I’m going to go, ‘What weight are you?’ Then I know you’ve only got certain 3 or 4 days then you’re going to have a shit time of it but it's your fault. I’m not their Dad, I’m their coach. I’ll give you every bit of advice and support I can but if you go out and eat KFC or you go out on the drink with your Mrs at the weekend and put loads of weight on, that's on you. You’ve got a responsibility to be on time. [P1]

Participants who had engaged with RWL practices noted that they had done so entirely without medical support. Outside of an inevitable lack of health monitoring, a consequence of this was the commonplace use of prospectively unreliable sources – such as social media or magazines – to inform weight-cutting practices.

I have searched online like ways to weight cut and stuff, or ways to water cut. I even tried saying ‘How much in pounds or kilos do you lose like every half-hour in a sauna?’, and there isn't a formula ‘cos it obviously depends on your body weight. [P3]

Young lads think that … if they read an article where a certain fighter has cut weight that way, they can do the same at home, but this guy will have done this with a team of professionals around him. This guy's in his bedroom or his living room or his bathroom doing this and there's no supervision, there's no medical guidance, they just do, ‘Oh yeah well X did this, oh fuck it, I’ll do that as well’. [P1]

[The competitor] nearly passed out twice, so if somebody is doing it on their own, doesn't have the right support, doesn't have somebody … some unskilled corner … massively dangerous. [P2]

3(b) Attributed impacts on health and everyday life

The influence of RWL on health was often expressed in terms of its impact on performance.

I’ve dehydrated myself down to get to there then on the day my energy levels are down, my strength's down, my focus is down, my cardio is affected by it. [P1]

Participants recurrently drew attention, however, to the fact that RWL does not take place in a social vacuum. Rather, fighters at this level needed to manage their usual vocational and social responsibilities concurrent to their weight-cutting, with further embedded risks.

You can't go and work on a construction site and then go and weigh-in in the evening ‘cos that's just not safe. [P3]

My friend was driving, and he was weighing-in at the same time, so he was driving when he’d not had much to drink, nothing to eat, so he was knackered when he was driving. [P4]

It was noted that, because fighters generally feel that responsibility for managing weight-cutting is theirs alone, they would often not seek help in their everyday lives – or even mention – if they felt unwell.

Like, I felt crap, but I didn't want to say anything ‘cos I didn't want to make everyone else at my work feel bad, because it's my choice to compete and it's not theirs. [P3]

Participants’ accounts of the impacts of RWL on fighters’ health were revealing on several levels, not least in terms of how they informed sense-making around risks and risk-minimisation.

I’ve not done it so far that it’d be dangerous, but I’ve known of people who have … I’ve seen people that just look inebriated. [P4]

It was common for participants to document the dangers of specific practices, such as hot salt baths, water-loading or taking fat burners.

You can wear sauna suits, hot baths … Hot salt baths, I’ve done hot salt baths. They work 100%. They’re as dangerous as shit but they work … I know people who have passed out. [P1]

I’ve never passed out. I’ve never known anyone to pass out, but a few people have been close … What you do is like a week before fighting, you do a thing called water loading … Then basically you have no water in you. [P7]

People will use steroids and certain banned substances to help get their weight down … but it's really dangerous. [P1]

However, and as evident above, the risks were often taken to be subordinate concerns to the RWL-related efficacy of a practice. Moreover, acknowledging that RWL practices could be associated with high risk, including the risk of death in the most extreme instances, did not mean that participants necessarily considered *themselves* to be at risk:

You’ve got to think how many people are weight-cutting. I know hundreds of friends in Thailand who are fighting. Only one of them's died. It's like one person out of hundreds of fighters has died. It's obviously not a problem … I’ve cut weight many times and I’ve never died, I’m still here. [P7]

4. Subverting RWL control(s)

Participants’ experiences of measures to restrict or reduce RWL practices were central, with particular emphasis on attempts to undermine/subvert the implication or efficacy of such controls.

Participants widely noted that even outright banned practices in most combat sports are only as effective as the detection systems in place, and these systems are typically confined to the highest levels. This was viewed as a clear obstacle to regulating many RWL practices in Thai boxing from the outset, though some participants did remain positive about the feasibility of some such controls.

I see on a Facebook post one mentioned rapid weight reduction steroids … They’re banned from U.S. Anti-Doping Agency (USADA), for Ultimate Fighting Championship (UFC) … Unless you’re fighting where they have stringent tests then you can take anything. [P1]

I’ve heard about some [ideas] like same-day weigh-ins. There's a lot of shows that do them anyway, and there you’ve got little chance because you can't rehydrate in an hour or two. I think they’re a good idea. [P3]

However, it was also noted that measures to control or restrict RWL could have the potential to exacerbate RWL. For example, discussing a potential weight-management system which aims to monitor and limit weight loss, P7 commented:

You could do a programme where you weigh-in three or two weeks before or a week before … if I did do that, I’d just tell them I want to fight at 75 and I’ll just weight cut for three weeks.

Participants also reported practical issues with introducing controls, for example with hydration testing:

[T]he other thing is the practical reasons behind it. I mean, if somebody's really not been drinking anything for a few days or not been drinking much for a few days, are they even going to be able to provide a sample. So, they might just flat out refuse to give one, or they might just turn down the fight because they don't want to do it and that's going to look bad for the promoters and I don't know if the promoters would be on board with that. [P3]

The reluctance of promoters to introduce controls was further noted by P5:

[T]he thing is, what they’ll do is if they’ve got someone who's light and someone who's heavy, they’re both brilliant and it’d be good money to get them to fight, they will say, ‘I will give you this much money if you fight her’ and then they will do anything they can to their body to get to that weight to fight them because it's for the money, isn't it at the top level?

The notion that the level of competition impacts attitudes towards RWL controls was also addressed by P4, who described responses to an event that planned to have an ‘on the day’ weigh-in:

They’ve already had responses from loads of clubs saying: ‘No we won't do it’, because they don't like the fact that they might have to fight at a more natural weight for them. Lots of elite clubs, the ones that collect belts and have hundreds of belts on the wall, a lot of them have said they just don't feel like they could send guys or girls to a show like that unless they were newbies or it would have to be like an N-Class show which is like novice, which is the lowest class of people. I think we’ve already spoken about it and my coach has already said that she thinks that the main event would have to be B-Class; she wouldn't be able to get an A-Class fight on the day. I don't know if you know but it's N for novice, which is no elbows and knees to the head and you wear shin pads. C-Class is no elbows and knees to the head but without shin pads. B-Class is knees to the head and A-Class is knees and elbows to the head; obviously no shin pads for C, B or A. So, she thinks that only B-Class fighters would be interested at the most to do a show like that. C-Class as well and N-Class would be fine as well just because they’re novices, but the highest level she thinks that no one would do it.

However, other participants were confident that participants at nominally lower levels of the sport would similarly engage in RWL measures:

I know guys who have taken water tablets; I know guys who have taken fat burners, and this is something simple as a Jiu Jitsu comp on a Saturday afternoon in a sports centre, this isn't even getting paid money, you pay to enter. So, these are the extremes that people are going to compete. Just for a hobby. [P1]

## Discussion

This study sought to identify social influences and processes that encourage and facilitate rapid weight loss in combat sports. Four themes were identified within the participants’ accounts which, in combination, described how RWL was learned, rewarded, magnified, often unmitigated, and inherently difficult to restrict. Participants maintained that it is arguably an inevitable consequence of the business of weight-setting itself. The evidence presented, moreover, underscores how RWL is prospectively widespread at all levels within the combat sport community, and that despite robust research that raises concerns about the dangers of the practice (Artioli et al., 2016; Lakicevic et al., 2022), the practice does not appear to be abating.

In attributional terms, all participants described RWL as essential for gaining maximum advantage, or at least avoiding being disadvantaged, when being matched with a nominally heavier and thus inferably stronger opponent. It was, however, typically understood in all given accounts that where both fighters are engaging in RWL, there is prospectively a net-zero gain. This dynamic is reminiscent of the ‘prisoner's dilemma’ that has similarly been explored with respect to the use of performance-enhancing drugs in sports (Chirico et al., 2021; Petróczi & Aidman, 2008). In short, when in any doubt, one must assume that one's competitor must be taking any advantage possible. This assumption, in turn, legitimates any action necessary to level an inferred playing field. As it is with drug-use, thus, any dangers around RWL can typically become a secondary concern to the business of winning at any cost.

Within the cultural terms described, it was also notable that – for some participants at least – ‘competitive dieting’ had a critical status of its own. Indeed, meeting weight goals alone was often seen as an achievement in itself, for demonstration to peers alongside fighting outcomes. Such self-concordance goals align with the ‘identified regulation’ component of Self-Determination Theory, and can be powerful drivers of behaviour and sense of accomplishment in themselves (Waaler et al., 2013). It was apparent that some fighters took active pride in the toughness/pain required to endure the RWL process to completion, given the alignment of such endurance with an understood place within the culture of Muay Thai. Statements referring to the ‘buzz’ that was experienced when achieving a weight target were numerous, and emphasised how those individuals strived, regardless of any detriments, to continue until their goals were met, potentially leading to increasing risk-taking behaviours (Woodman et al., 2013). This commitment-focus within Muay Thai was further underscored by participants’ descriptions of how they would support peers to persist with RWL even if they were unwell, because their value as a fighter was inextricably linked to grit and adherence, and to the cultural dedication they had made (Aloui et al., 2013; Aloui et al., 2016; Franchini et al., 2012). Such findings also echo reports of ‘team doctoring’, which emphasise the ways in which medical advice is passed on by peers irrespective of professional skills and knowledge (Al-Hashmi & Matthews, 2022).

Psychological processes in learning ‘what to do’ were recurrently described by participants in this sample. In particular, focus fell upon when they sought information, what they looked for, and which sources they used to inform themselves. Messages are known to be highly persuasive when source credibility is seen as being high, for example through perceptions of expertise or celebrity status (Pornpitakpan, 2004). Such effects were highly apparent in our sample, potentially more so in the absence of guidance from their trusted, known sources (Artioli et al., 2010; Finlay et al., 2022; Matthews et al., 2019).

Findings in the current study also align with previous research, reporting that a focus on performance does not encourage fighters to consider the consequences of actions on well-being (Al-Hashmi & Matthews, 2022); participant accounts reveal that goal mindset was a driver for making weight. Further, these results demonstrate that self-directed preparation for the contest had the potential to magnify RWL behaviours, particularly when gradual weight loss was not managed, and this subsequently increased the extent of the weight loss required. Participant testimony describes the lack of support fighters have while making weight and indicates that, subsequently, they may take more risks and receive less help in the event of illness. Past research similarly emphasises the key role of professional health support in highlighting health consequences to fighters (Follmer et al., 2020), and that RWL has the potential to be reduced in competitors who have the support of a trained dietitian (Park et al., 2019).

Mitigation of the impacts of RWL has been highlighted by the current study as an important area for future research. Participants revealed that the negative consequences associated with their RWL behaviours may be underplayed, meaning fighters may not engage in help-seeking behaviour. This is a finding which aligns with previous reports that even athletes with an understanding of health-related repercussions may continue activities when unwell (Malcolm et al., 2024). Furthermore, participants noted that risks from RWL behaviours may be widened through undertaking everyday activities (e.g. driving) whilst experiencing cognitive or physical impairment.

It is important to highlight that differences in risk perception were notable among participants. Some actively highlighted threats to health, and the confusing or even contradictory issues in practising RWL within a ‘hobby’. Others, however, were keen to downplay the personal risks posed by RWL, commonly citing the ubiquity and success of the practices (i.e. normalising them), and the relatively low rates of death or serious illness reported in a sport with high participation. The latter prospectively speaks to fighters’ reliance on an availability heuristic (Miller et al., 2012; Waters et al., 2023) in making such risk-related judgements. This metacognitive ‘shortcut’ describes the way individuals will often weigh the most familiar and/or most recently accessed information disproportionately highly when undertaking situational assessments about the accuracy of statements and the efficacy of courses of action (Pan et al., 2021). As noted by a number of participants above, fighters in the non-professional sphere are rather more likely to glean their information about RWL via easily accessible online materials than from more reputable medical/scientific sources or agents. Moreover, individuals’ selection of online materials around health matters has itself been demonstrated to show a routine confirmation bias (Meppelink et al., 2019); i.e. the materials likely to be most frequently (and recently) accessed by fighters who are already committed to RWL are those that do not challenge this central view, but rather reinforce it. In this way, being a part of Thai boxing culture and downplaying the health risks of RWL to self can, for some at least, become something of a self-perpetuating state.

### Study limitations

Sample size and general representativeness are not key concerns for qualitative work; sample size is not indicative of the amount of data the sample holds (Malterud et al., 2016). Information power in this data set is strong due to (1) the high-quality rich dialogue and clear communication of participants detailing complex and contexualised experiences, and (2) the participants reporting experiences that have not previously been described in the research literature. Nonetheless, it should be noted that the nominally small sample in this study is a direct output of researching a ‘hard to access’ group of confidants. The findings above address a matter (RWL) that could have implications for Muay Thai in reputational terms, and thereby negatively influence future uptake at the very least. While the study initially sought to engage a larger number of participants, those who did engage were they clear that they did so under the highest levels of confidentiality. In these terms, the findings of the analysis might be viewed in the same light as many other qualitative studies of inherently ‘delicate’ matters (see Hopkins & Miller, 2023), i.e. *the best we can reasonably hope for given the circumstances*. Every effort has been made, however, to fully contextualise the findings such that they might be useful in future research.

A secondary limitation relates to the absence above of matters pertinent to gender, race, and participant identity in general (beyond the key issues of experience). While this is chiefly an outcome of ethical requirements regarding reporting, it does render relevant future research that might focus more explicitly on how different people might engage with RWL.

## Conclusion

Participants argued that RWL did give a fighter a competitive advantage and that those at all levels of the sport benefit from the practice, as fight ‘wins’ demonstrate the credentials of coaches and clubs as well as the fighters. Therefore, even fighters who do not agree with RWL practice are encouraged to engage with it. Normative beliefs are noted as core influences of intentions and behaviours (Junker et al., 2022), and this emphasises the centrality of social norms within decision-making. This centrally highlights how interventions to limit behaviours would need to be procedural and behavioural, rather than simply attitudinal.

The findings of this study highlight the challenges of introducing controls to prevent or reduce RWL. Participants maintained that, although RWL behaviours may be technically ‘against the rules,’ the lack of measures to apply said rules means that there were no sanctions for engaging in RWL. As a result, arguably at lower-classification contests where there are fewer mechanisms to identify practices, RWL may be more intense, even though the material rewards of winning are less. Our findings align with doping literature which notes that doping is more likely when it is associated with career advancement, endorsement by peers, and a low chance of detection or penalties (Ring et al., 2018). Furthermore, participants in our study highlight that the introduction of mechanisms to reduce the extent of RWL may counterproductively extend duration (if weight were monitored for longer and fighters responded by engaging in RWL throughout this period) or exacerbate impact (if the time between weigh-in and bout were reduced thus restricting rehydration attempts). The lack of controls at low levels of sport highlights the importance of context in understanding health behaviours. Any proposed RWL intervention would need to address how able combat sport clubs are to operationalise controls; socioeconomic factors present a persistent challenge in both implementation of, and access to, health services (Braveman & Gottlieb, 2014). Grassroots clubs can be assumed to have little or no resource to employ professional services or adopt high-tech solutions to addressing RWL.

Overall, the findings in this study describe how RWL is socially embedded within Muay Thai; it is a strength of this paper that it helps to develop a theoretical understanding of RWL from the perspective of the social processes. Although previous (largely quantitative) research has explored the performance-related impacts of RWL, such work does not describe the factors which impact the learning of the practice, increase the extent or duration of RWL behaviours, encourage or prevent help-seeking, or aide the implementation of measures to restrict practice. Furthermore, previous qualitative work had chosen to use elite athletes (Pettersson et al., 2013) and had noted that weight regulation behaviour was potentially likely to be less aggressive in lower levels of the sport; we chose to use a non-professional sample where participant testimony suggests that this is not the case. We recruited a stratified sample to provide a rich detailed account of RWL across different roles in Muay Thai, Future research should give greater emphasis to RWL at lower levels of the sport as these fighters may be using aggressive RWL methods without support, and the role of coaching ‘power’ in this, both as a model of protection and harm.

## Data Availability

The data generated during and/or analysed during the current study are not publicly available nor are they available on request due to original conditions of ethical approval, which note the high likelihood for participant identification within the community of focus should full transcripts be released, even though those transcripts are fully redacted of names, places, exact dates and so forth.
